# An Observation Concerning Catarrhal Inflammation of the Eustachian Tube

**Published:** 1881-11

**Authors:** Lucien Howe


### An Observation Concerning Catarrhal Inflammation of the Eustachian Tube.

By Dr. Lucien Howe.

In order to present a distinct mental picture of the affection, concerning which, these few remarks are made, it seems well to begin by sketching the history of a case which illustrates its principal clinical features.

Such examples are not difficult to find, and I select one which is especially appropriate on account of a certain peculiarity to be mentioned later. This patient was a girl of about sixteen, whom I first saw on the 15th of last May. Being a person of unusual intelligence, her history was stated with clearness and exactness, somewhat as follows: A few days previously she began to suffer pain in the vicinity of the left ear, and as an uncomfortable sensation extended downward in the direction of the mouth, the trouble was at first attributed to a carious tooth. The fallacy of this supposition was made evident not only by the pain becoming subsequently more localized, but also by the fact that after the extraction of the suspected tooth, the pain again appeared. This was the only subjective symptom complained of, and the objective signs, with a single exception, were equally meagre and unsatisfactory. An inspection of the membrani tympani showed it to be slightly concave, but otherwise normal, and on pressing the air through the Eustachian tube with a Politzer bag, or by holding the nostrils closed during forcible expiration according to Valsava's method of inflation, a distinctly moist rale was heard by means of the auscultation tube. These few ill-defined symptoms apparently indicated some trouble, rather difficult to locate exactly, and of no very great importance after it was diagnosed. More than one such patient applying to a routine practitioner receives a prescription for "ear drops" containing opium, and is dismissed from the office and from his thoughts. There was, however, a peculiarity about this case, which not only made it interesting in itself but which lead to considerations that were deemed worthy of notice.

This peculiarity, consisted in a cleft in the palate, which was very broad posteriorily and extended forward almost to the alveolar process. An artificial palate of hard rubber had been ingeniously arranged to partly cover the defect, but on removing that, an uninterrupted view could be obtained of all the important tissues about the posterior nares and upper part of the pharynx, reminding one of the illustrations to be found in

Cohen* and elsewhere. The opening of the Eustachian tube on each side was shown with a distinctness which could never have been gained with a mirror, and its condition could be observed at leisure without any of the inconveniences of rhinoscopy. On examining the opening of the tube on the left side, it was seen to differ materially from its fellow. The mucous membrane in its vicinity, when first seen, was red and œdematous, and from the partially closed orifice a whitish clot of mucous was oozing.

The case was therefore one well worthy of unusual study—a distinct catarrhal condition of the Eustachian tube presented for direct inspection.

This disease is not generally considered a common one, and to find it associated with cleft palate is still more rare.

Most writers on the ear, by omission or otherwise, lead the student to think that catarrh of the Eustachian tube is a rare disease. Of American and English authors this is especially true. When speaking of a catarrhal condition of the middle ear, Gruber† simply mentions the fact that the tube may be "abnormally constricted, or closed, when there is but little of the inflammatory products in the tympanic cavity." Schwartze‡ describes the post-mortem appearances in such a condition, and Von Troeltsch|| has also dwelt upon the pathological changes in aggravated forms.

But one of the clearest chapters upon the subject is found in the excellent work recently published by Urbantschitsch,§ who describes in detail the different symptoms accompanying a catarrh of the Eustachian tube. These are pains more or less localized, a moist rale heard through the diagnostic tube, with the rhinoscope the orifice of the tube appears swollen, and when sounded, it feels constricted. It is undoubtedly true that this condition is often found with catarrh of the middle ear, but

* Diseases of the Throat, page 185.

† Lehrbuch der Ohrenheilkunde, s. 448.

‡ The Pathological Anatomy of the Ear, p. 137.

|| Archiv fuer Ohrenheilkunde IV, s. 136.

§ Lehrbuch der Ohrenheilkunde, s. 240.

there seems good authority for considering that the pain in, or about the ear, so commonly experienced, may be due to such an abnormal condition of the Eustachian tube alone.

But the general practitioner is not always provided with a diagnostic tube, a rhinoscope, a catheter or sound, or even if he is skilled in the refinements of diagnosis, it is sometimes necessary to attempt relief for a pain in the vicinity of the ear, under circumstances which make a thorough examination of the conditions impossible. In such a case it is, therefore, in the highest degree necessary to keep in mind the fact that the pain may be due to a morbid process going on in the Eustachian tube, and this should be treated, instead of resorting at once to "ear drops" of questionable value. This brings us to the question of the proper treatment of such a condition. The subject is of course too large for discussion in a short paper, and most of the text books treat of it, even though indirectly, as associated with catarrh of the middle ear. As one of the first means to be tried, we usually find it recommended to inflate the ears by means of Valsava's or Politzer's methods. Undoubtedly this may be advisable in many cases, but I am inclined to think that local application of astringents, especially tannin or alum, in weak solutions, with the atomizer, or a gargle of similar character, will do more good than the simple opening of the tube by inflation. These two suggestions as to the frequency, and as to the treatment, of catarrh of the Eustachian tube seemed to be worthy of note in connection with the case cited.

